# Esophageal Atresia and Tracheoesophageal Fistula with Unilateral Pulmonary Agenesis - Hypoplasia

**Published:** 2013-04-01

**Authors:** Shraddha Verma, Jai Kumar Mahajan, Katragadda Laxmi Narsimha Rao

**Affiliations:** Department of Pediatric Surgery, PGIMER, Chandigarh, India

**Keywords:** Pulmonary agenesis, Tracheoesophageal fistula, Esophageal atresia

## Abstract

Association of unilateral severe pulmonary hypoplasia or agenesis and esophageal atresia (EA) with or without tracheoesophageal fistula (TEF) is an exceedingly rare and highly lethal combination. We report a case of full term male baby who had EA with TEF and right lung hypoplasia, managed at our centre. He is alive and doing well at 10 years of age.

## INTRODUCTION

The combination of tracheoesophageal and pulmonary malformations is unusual and reportedly carries a high mortality. Association of unilateral severe pulmonary hypoplasia or agenesis and esophageal atresia (EA) with or without tracheoesophageal fistula (TEF) is an exceedingly rare and highly lethal combination [1]. The longest survivors that have been reported were 5 years old at the time of report [2, 3]. Herein, we describe one such case that is doing well 10 years after the surgical intervention.

## CASE REPORT

A full term, 2.0 Kg male baby, delivered at home presented with excessive frothing within a few hours of birth. The antenatal period was unsupervised and the delivery uncomplicated. At presentation, the baby had mild respiratory distress. The orogastric tube could not be passed beyond 10 cm from the lower lip. Breath sounds were not audible over right hemithorax. The chest x-ray revealed opacification of the right hemithorax with a mediastinal shift. The red rubber catheter was seen in the upper esophageal pouch at the level of T1 vertebra and the abdominal gas pattern was normal. 

The baby underwent right postero-lateral thoracotomy. The lung tissue was not seen in right hemithorax. The gap between the upper and the lower pouch was approximately 4cm. The baby underwent fistula ligation, right cervical esophagostomy and gastrostomy. The baby was discharged on gastrostomy feeds on 10th post-operative day. At 6 months follow up, a chest x-ray showed hyperinflation on the left side and mediastinal shift to the right. 

**Figure:**
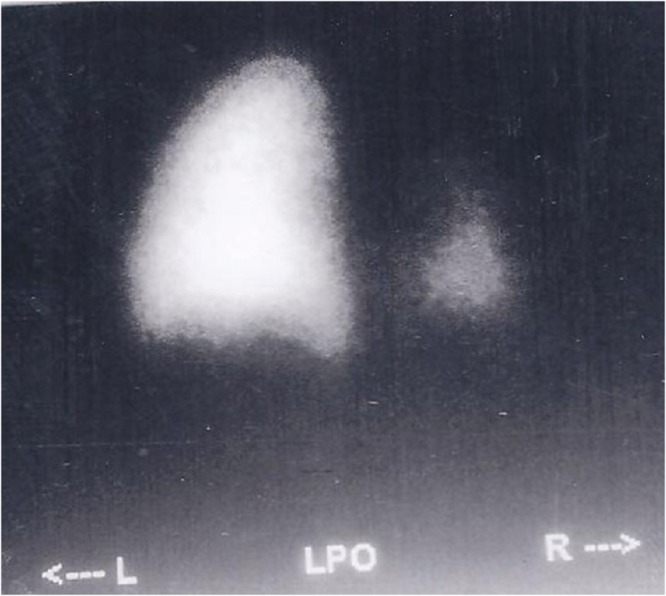
Figure 1: Ventilation perfusion scan with normal left lung and hypoplastic lower lobe of right lung.

Bronchoscopy, performed at 6 months of life, revealed absence of the right main bronchus and the trachea continued into a single bronchus with branching seen in the distal part. Ventilation perfusion scan, at 6 months of life, of the lung showed normal left lung and hypoplastic lower lobe on right side (Fig. 1). Echocardiography, done at 7 months, showed a hypoplastic right pulmonary artery. The patient underwent fundal tube esophagoplasty [4] at one year of age. 

At 10 years of age, the child is feeding well, has occasional regurgitation and no respiratory distress while playing. His growth parameters correspond to the centiles. When last seen a year ago, he had achieved normal developmental mile stones with normal intelligence. However, the chest was asymmetrical with depression on the right side with scoliosis and concavity to the right.

## DISCUSSION

Morgagni in 1762 first described congenital underdevelopment of the lungs. Since then several cases have been reported in literature [1-3, 5-15]. This entity is believed to be caused by failure of the developmental balance between the two lung buds at approximately 4th week of gestation due to unknown aetiology [16]. Congenital underdevelopment of the lungs was classified by Schneider as (1) Agenesis: the complete absence of the carina and the main bronchus, the lung, and the pulmonary vasculature; (2) Aplasia: the carina and the rudimentary bronchus are present, the pulmonary vessels and the alveolar tissue are absent; and (3) Hypoplasia: an ill-defined bronchus is capped by underdeveloped alveolar tissue. The incidence of unilateral pulmonary agenesis is one in every 10,000 to 15,000 autopsies. Almost 50% of the patients have associated anomalies of the other systems which include cardiac, diaphragm, lip and palate, genitourinary tract, vertebral and the radial.

The combination of TEF with severe pulmonary malformations had been universally fatal until 1985 when first documented case of long term survival was reported [2]. Since then, fourteen patients with survival beyond newborn period have been reported [1-3, 5-9, 11-14]. 

All 14 survivors have agenesis or hypoplasia of the right side (Table 1). Two patients underwent cervical esophagostomy and gastrostomy. Primary esophagoesophageal anastomosis could be done in other patients. Associated anomalies included IHPS, duodenal atresia, imperforate anus, right sided aortic arch, tracheal stenosis, rib and vertebral anomalies.

**Figure:**
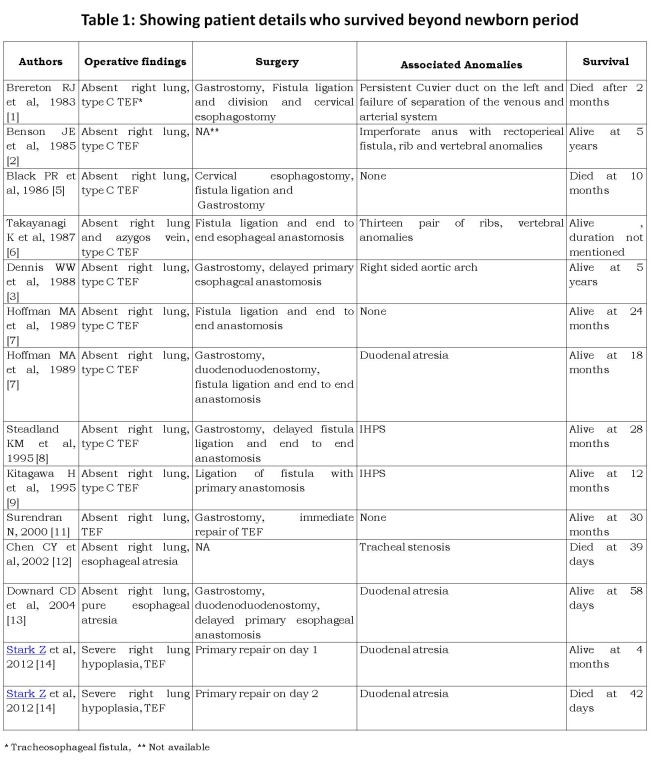
Table 1

Hoffman et al described two neonates who underwent primary repair shortly after birth [7]. According to them, early protection and preservation of respiratory units should be the prime goal in management of these patients and is best feasible by primary repair of the tracheoesophageal lesion. Steadland et al [8] suggested that the gestational age, size, and the associated medical problems need to be considered when planning therapy in babies with associated tracheobronchial anomalies. During surgery, retraction of the heart is required for exposure, resulting in frequent episodes of hypotension and bradycardia. Death in these patients is due to progressive respiratory failure. Hence, preservation of the respiratory function should be the goal, even if esophageal continuity needs to be sacrificed.

Based on clinical signs and chest skiagram, associated severe pulmonary malformations may be suspected in patients of EA and TEF preoperatively. Bronchoscopy can be performed to know presence of bronchus and its site of origin, and if performed preoperatively, can also locate the fistulous opening.

## Footnotes

**Source of Support:** Nil

**Conflict of Interest:** None
